# Transstadial Transmission and Replication Kinetics of West Nile Virus Lineage 1 in Laboratory Reared *Ixodes ricinus* Ticks

**DOI:** 10.3390/pathogens9100780

**Published:** 2020-09-24

**Authors:** Cristian Răileanu, Oliver Tauchmann, Ana Vasić, Ulrike Neumann, Birke Andrea Tews, Cornelia Silaghi

**Affiliations:** 1 Institute of Infectology, Friedrich-Loeffler-Institut, Suedufer 10, 17493 Greifswald-Insel Riems, Germany; cristian.raileanu@fli.de (C.R.); oliver.tauchmann@fli.de (O.T.); ana.vasic@fli.de (A.V.); ulrike.neumann@fli.de (U.N.); birke.tews@fli.de (B.A.T.); 2 Institute for Medical Research, University of Belgrade, Dr. Subotica 4, 11129 Belgrade, Serbia; 3 Department of Biology, University of Greifswald, Domstraße 11, 17489 Greifswald, Germany

**Keywords:** West Nile virus, *Ixodes ricinus*, transstadial persistence, arbovirus

## Abstract

West Nile virus (WNV) is a mosquito-borne agent that has also been isolated from several tick species. Vector competence of *Ixodes ricinus*, one of the most common tick species in Europe, has been poorly investigated for WNV to date. As such, to evaluate the vector competence, laboratory reared *Ixodes ricinus* nymphs were in vitro fed with WNV lineage 1 infectious blood, allowed to molt, and the resulting females artificially fed to study the virus transmission. Furthermore, we studied the kinetics of WNV replication in ticks after infecting nymphs using an automatic injector. Active replication of WNV was detected in injected nymphs from day 7 post-infection until 28 dpi. In the nymphs infected by artificial feeding, the transstadial transmission of WNV was confirmed molecularly in 46.7% of males, while virus transmission during in vitro feeding of *I. ricinus* females originating from infected nymphs was not registered. The long persistence of WNV in *I. ricinus* ticks did not correlate with the transmission of the virus and it is unlikely that *I. ricinus* represents a competent vector. However, there is a potential reservoir role that this tick species can play, with hosts potentially acquiring the viral agent after ingesting the infected ticks.

## 1. Introduction

West Nile virus (WNV) is a zoonotic arbovirus with a recognised worldwide dispersal causing neurological diseases in animals and humans. Since its first isolation from the blood of a febrile woman in the West Nile district of Uganda in 1937, WNV has extensively propagated, and is at present endemic in all continents apart from Antarctica [1]. The virus belongs to the *Flavivirus* genus alongside more than 70 viruses, the majority being transmitted by mosquitoes and ticks. WNV is maintained in an enzootic transmission cycle between ornithophilic mosquitoes (the most important representative of which is *Culex pipiens*) and avian species that act as amplifying hosts [1,2].

WNV has also been isolated from several species of ixodid (hard) and argasid (soft) ticks from endemic areas of Asia, Europe and Africa [3,4,5,6,7], suggesting a potential implication of these vectors in the transmission cycle of WNV. In addition, different tick species have been experimentally infected with WNV, revealing the ability of ticks to acquire the virus from infected animals and to transmit it to the next developmental stage. As such, *Amblyomma tigrinum*, *A. ovale* and *A. tonelliae* larvae successfully acquired the virus after feeding on viremic chicks. Furthermore, unfed nymphs of *A. ovale* and *A. tonelliae* maintained WNV transstadially, being positive for the virus after plaque assay. In the same study, infection of *A. tigritum* nymphs after feeding on WNV-infected chicks was also successful while the virus persisted in the adult stage at a low rate (3.7%) [8]. WNV transstadial persistence was previously confirmed by recovering the virus from Vero cells and by RT-qPCR also in *Ixodes scapularis*, *Dermacentor andersoni* and *D. variabilis* after the feeding of larvae and nymphs on infected mice and hamsters [9]. *Ixodes pacificus* is another ixodid tick species for which maintenance of WNV from larval stage to nymph was indicated by RT-PCR and plaque assay after infecting larvae by feeding on viremic song sparrows [10]. None of the above-mentioned tick species showed vector competence for the viral agent, with the tick-to-host transmission being surveyed by feeding ticks on naïve hosts.

In contrast, *Hyalomma marginatum* ticks, experimentally infected with WNV by feeding on viremic rabbits and confirmed positive by RT-PCR, showed potential to transmit the virus to a naïve host after maintaining the agent through the molting process [11]. Infected nymphs and females that were exposed to the virus at the stages of larvae and nymphs, respectively, transferred the virus to uninfected rabbits during feeding, with the tick-infested rabbits developing WNV antibodies. Soft tick *Ornithodorus moubata* was also able to successfully transmit WNV to naïve hosts (mice and day-old chicks) under experimental conditions [12,13].

In order to determine the vector competence of *Ixodes ricinus* for WNV, one previous study experimentally infected the nymphal stage of this species by placing ticks to feed on infected BALB/c mice [12]. The successful infection of ticks was confirmed by RT-PCR at two days after engorgement, while after 28 days the ticks tested negative. The authors concluded that infected nymphs of this tick species are not competent vectors for WNV since no replication of the virus was observed.

*Ixodes ricinus* has the most widespread geographic distribution in Europe [14]. This tick species is able to feed on over 300 different vertebrate species and it is involved in the transmission of various bacterial, viral and protozoan pathogens of medical and veterinary importance [15,16,17]. The main agents transmitted by *I. ricinus* are *Borrelia burgdorferi* sensu lato (Lyme disease agent) and the Western European subtype of tick-borne encephalitis virus [18,19], a member of the *Flavivirus* genus closely related to WNV [20]. *Ixodes ricinus* immature stages frequently parasitize birds [21], the natural hosts for WNV, leading to a potential acquisition, transstadial maintenance and transmission of the virus to naïve hosts during blood feeding.

The WNV outbreaks in recent years have been described in countries from Southern and South-eastern Europe but also registered an expansion to countries from Central Europe [22]. The climatic conditions (long and dry summer seasons) favoured WNV transmission, notably during the 2018 season, during which WNV occurred in more countries than in previous years [23]. Germany also registered the first autochthonous cases of WNV in birds in 2018. Since then, Germany reported several WNV cases in birds, horses and humans, indicating, as such, the expansion of the virus in Europe from the south to the north [24].

The spread of WNV virus towards regions in which *I. ricinus* ticks are abundant [25] corroborated with the repeated isolation of the virus in several tick species, and the limited experimental investigations for WNV in *I. ricinus* represented the arguments for undertaking a vector competence study using laboratory reared *I. ricinus* ticks and WNV lineage 1. Therefore, the aims of this study were to explore (i) the kinetics of WNV lineage 1 replication in *Ixodes ricinus* nymphs (ii) the transstadial transmission of WNV from nymphs to adults and (iii) the potential transmission of WNV by *Ixodes ricinus* females during blood feeding.

## 2. Results

### 2.1. WNV RNA Kinetics in Ixodes ricinus Injected Nymphs

**Figure 1:**
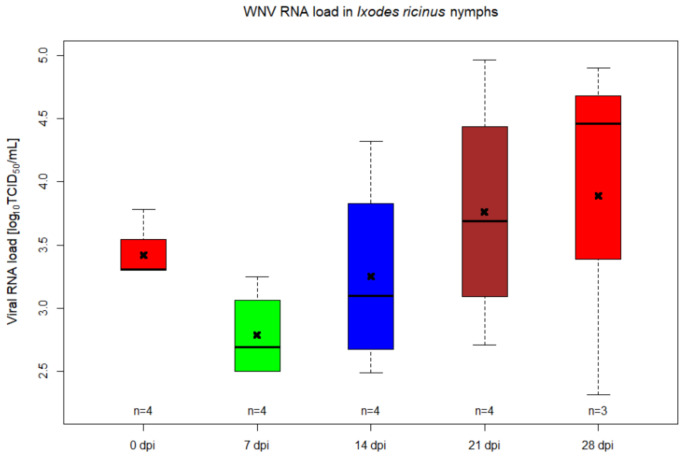
West Nile virus (WNV) titre in *Ixodes ricinus* nymphs injected by percoxal route at different timepoints. The horizontal black lines represent the medians, the x indicates the mean, the boxes show the interquartile ranges and the whiskers indicate the minimum and maximum values. The number of samples tested per timepoint is indicated above the *x*-axis.

**Figure 2:**
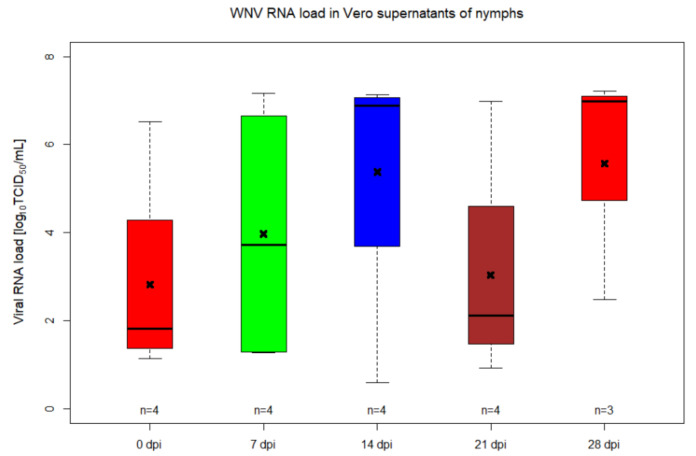
WNV titre in supernatants of Vero cells inoculatd with homogenates of *Ixodes ricinus* nymphs injected by percoxal route at different timepoints. The horizontal black lines represent the medians, the x indicates the mean, the boxes show the interquartile ranges and the whiskers indicate the minimum and maximum values. The number of samples tested per timepoint is indicated above the *x*-axis.

A percoxal route for infection of ticks with WNV lineage 1 was used in order to follow the replication kinetics of the virus at several different timepoints. As such, injected *I. ricinus* nymphs were collected at different days post-infection (days 0, 7, 14, 21 and 28) and tested for their virus load by RT-qPCR and by inoculating tick homogenates on Vero cell monolayers for confirmation of infectious viral particles. Out of 98 nymphs injected with WNV, 79 (80.6%) died within the first seven days post-infection, while from the control group, five out of 12 (41.7%) nymphs died within the same interval. The 19 surviving ticks of the WNV injected group were collected as mentioned above (four ticks per collection point—0 dpi until 21 dpi, three ticks at 28 dpi) and WNV RNA load was relatively quantified by RT-qPCR. All tick homogenates and Vero supernatants collected at day 7 after inoculation were confirmed positive for WNV RNA (Figure 1 and Figure 2, Table S1). The mean WNV RNA load in nymphs from 0 dpi was 3.42 log_10_TCID50/mL, registering a statistically significant drop until 7 dpi (2.79 log_10_TCID50/mL) (*p* = 0.0249), followed by a continuous increase in RNA load until 28 dpi (3.89 log_10_TCID50/mL), indicating that WNV was injected percoxally, replicated during the incubation period in the *I. ricinus* nymphs (Figure 1, Table S1). At all timepoints, there is at least one sample with a much higher titre in the supernatant of Vero cells inoculated with the tick homogenate than in the original tick homogenate, showing the presence of infectious virus (Figure 2, Table S1).

### 2.2. Transstadial Transmission of WNV

**Figure 3:**
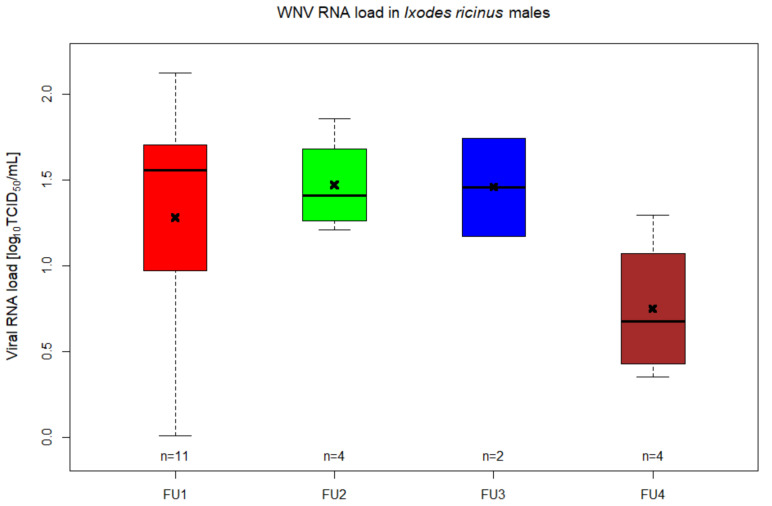
Viral RNA titres of WNV in *Ixodes ricinus* males. Different groups indicate the different FUs in which nymphs that molted into adult males were infected with WNV by in vitro feeding. The number of samples are indicated above the *x*-axis. Horizontal black lines are representing the medians, the x indicates the mean, the boxes show the interquartile ranges and the whiskers the minimum and maximum values.

**Figure 4:**
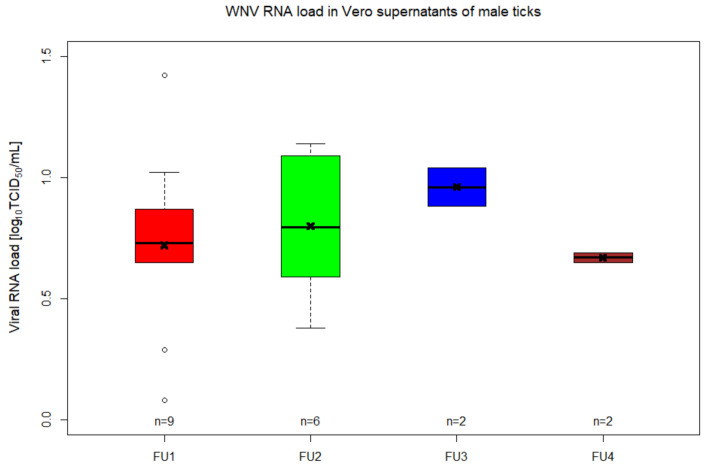
Viral RNA titres of WNV in Vero supernatants of *Ixodes ricinus* males. Different groups indicate the different feeding units (FUs) in which nymphs that molted into adult males were infected with WNV by in vitro feeding. The number of samples are indicated above the *x*-axis. Horizontal black lines are representing the medians, the x indicates the mean, the boxes show the interquartile ranges and the whiskers the minimum and maximum values. The values outside the interquartile ranges are represented as outliers.

The next step was to study the transstadial transmission of WNV from the nymphal immature stage to adult ticks. Artificial feeding of nymphs was done in five different feeding units (FU) (four FUs for the experimental group and one FU for control group) each having 30 nymphs and one female to stimulate attachment to the membrane. In total, the feeding lasted for 9 days, after which 48.3% (58/120) of nymphs from the experimental group fed until repletion (FU1 = 20; FU2 = 15; FU3 = 10; FU4 = 13), while in the control group, 43.3% (13/30) nymphs were engorged. Two engorged nymphs from the experimental group were collected right after repletion, being confirmed as positive for WNV RNA after RT-qPCR from both tick homogenates and Vero supernatants. In total, 56 engorged nymphs fed with infectious blood were incubated. The molting of nymphs into adult stage started 31 days from the completion of feeding, resulting in a molting rate of 94.6% (53/56; 30 males and 23 females). After processing each individual male tick, 14 samples (46.7%; 14/30) tested positive for WNV RNA in both types of samples (RNA from ticks and from Vero supernatants) (Figure 3 and Figure 4, Table S2). Relative quantification of WNV RNA from ticks showed viral loads that varied between 0.01 and 2.12 log_10_ TCID50/mL (Figure 3, Table S2). In addition to these samples that tested positive in both analyses, RNA samples from seven tick homogenates were positive for WNV after RT-qPCR, while another five samples showed amplification for WNV RNA only from Vero supernatants (Table S2).

### 2.3. Vector Competence of Ixodes ricinus Females

*Ixodes ricinus* females that were fed with WNV infectious blood meal at nymphal stage were placed in individual FUs in order to investigate the virus transmission during artificial feeding. In total, 23 FUs were used for the ticks that were fed on infectious blood meal as nymphs, while the control group was added in one FU (six females and seven males). Blood meals were collected at 24 h intervals starting from the second day of feeding for four consecutive days. During the collection of the blood samples, FUs were visualised under a stereomicroscope and the attachment of females to the membrane was noted. At day 1, eight females from the experimental group were feeding while, for the next days, eight, 11 and 13 females were observed to be feeding at days 2, 3 and 4, respectively (Table 1). After testing blood meals from all FUs for the presence of WNV RNA, no sample was found to be positive for the virus-specific target from either blood or Vero supernatants. Artificial feeding was carried out until all ticks detached from the membrane. After the in vitro feeding, all partially fed *I. ricinus* females and all males underwent a similar analysis course for the detection of WNV RNA (PCR on RNA extracted from the ticks and inoculation of Vero cells with tick homogenate and detection of viral RNA in the cell culture supernatant). The results showed two positive Vero supernatants of two female ticks partially fed, collected at day 3 and 4 of feeding and evaluated as dead at the time of collection, with a titre of 0.29 and 0.02 log_10_TCID50/mL, respectively.

**Figure 5:**
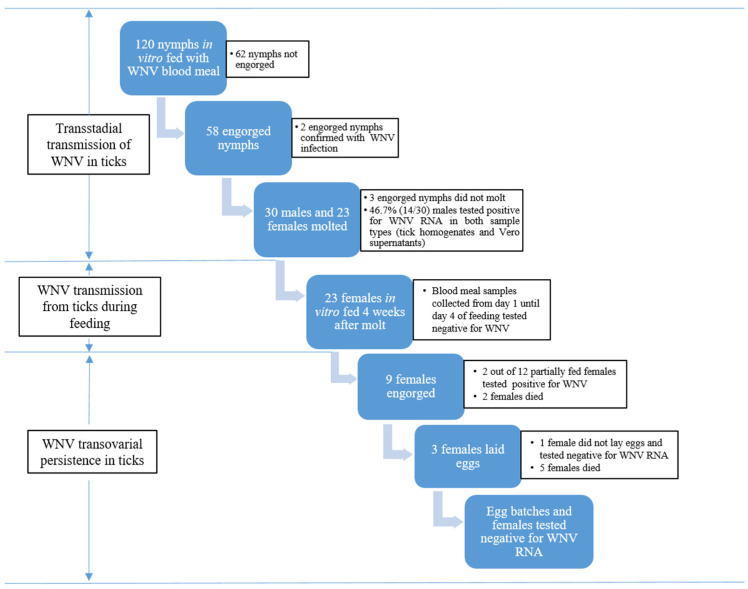
Experimental work for the vector competence study of *Ixodes ricinus* for WNV lineage 1.

In total, nine females from the experimental group and three from the control group fed until repletion and they were incubated to lay eggs (Figure 5, Table 1). During the incubation period, three females from the experimental group laid eggs, five died and one female was alive at the collection point (day 97 of incubation after the finalization of artificial feeding) (Figure 5, Table 1). Collected egg batches and female ticks tested negative for WNV RNA after RT-qPCR reaction.

All samples from the negative control groups were tested negative by RT-qPCR for WNV RNA in either tick homogenates, blood meals or Vero supernatants.

## 3. Discussion

The experimental work in this study was done to acquire new insights regarding the vector competence of *Ixodes ricinus* ticks for West Nile virus lineage 1. As such, ticks were successfully infected with WNV lineage 1 by percoxal injection and an in vitro feeding method was applied to also infect ticks and further on, to observe the virus transmission from infected ticks to the blood meal during feeding. The main findings of the study refer to (i) active replication of WNV in injected *I. ricinus* nymphs during 28 days of incubation, (ii) successful infection of nymphs with WNV by artificial feeding and (iii) transstadial persistence of the virus from the immature nymphal stage to the adult stage.

Our data showed that *I. ricinus* ticks infected parenterally with WNV support the active replication of the virus, this being observed in ticks incubated for 28 days post infection. The inoculation of pathogens by microinjection requires special attention as a high proportion of ticks can die due to the injection injuries [26]. After injection, we also noticed a high rate of mortality among ticks: 79 out of 98 nymphs died within the first seven days post-infection while, from the control group, five out of 12 nymphs died within the same interval. This method of inoculating the viral agent into the hemolymph of ticks does not represent the natural route and it bypasses the midgut barrier. Even though it does not mimic the infection of ticks under natural conditions, the experimental parenteral inoculation did provide evidence for WNV replication. The increase in the viral RNA amounts was observed from day 7 post-infection (7 dpi) until 28 dpi. Inoculation of the homogenate on Vero cells proved the presence of infectious virus, as several of these samples had a much higher genome copy number compared to the initial inoculum. A decrease in viral titres was observed from 0 dpi until 7 dpi. This decrease in viral load could represent the time frame during which WNV particles are penetrating the host cells and initiate the replication. In addition, tick immune system could also contribute to the observed initial decrease of WNV RNA. Similar data were registered for tick-borne encephalitis virus inoculated by percoxal route into females of a known vector tick species, namely *Dermacentor reticulatus*, but the decrease in RNA copies quantified by RT-qPCR was observed during the first 2 dpi [27].

By in vitro feeding with blood spiked with WNV lineage 1, *I. ricinus* nymphs were successfully infected and maintained the virus transstadially, with 46.7% of the tested males being positive. The presence of infectious virus in these samples could also be confirmed through the inoculation of Vero cells, which lead to increased titres in some samples and allowed viral detection from samples in which the direct detection had failed. These results contradict the findings of a previous study published by Lawrie et al. (2004), during which WNV was not able to replicate in *I. ricinus* tick species [12]. The authors observed this lack of viral replication after successfully infecting nymphs on viremic rodents and not finding evidence of WNV RNA by RT-PCR in ticks 30 days later.

Furthermore, the results of our study are in concordance with previous reports that indicated the transstadial persistence of WNV in hard ticks without any proof of viral transmission to the host during feeding [8,9,10]. A major difference between the current study and the existing published results of the experimental infection of ticks with WNV is represented by the method of infecting ticks. As such, we successfully infected *I. ricinus* nymphs using an in vitro feeding method while, in previous reports, infection of ticks was done by allowing them to feed on WNV-infected hosts. In these previous studies, several tick species acquired the virus from infected hosts: larvae and nymphs of *Amblyomma ovale*, *A. tigrinum*, and *A. tonelliae* after feeding on chicks [8], *Ixodes scapularis* larvae, larvae and nymphs of *Dermacentor andersoni* and *D. variabilis* from infected mice and hamsters [9], *Ixodes pacificus* larvae infected from viremic song sparrows [10], larval and nymphal stages of *Hyalomma marginatum* from rabbits [11], second instar of *Ornithodorus moubata* and *I. ricinus* nymphs after feeding on mice [12]. We demonstrated that artificial feeding is a suitable method for infecting ticks and could be utilized in various experimental studies that involve tick–pathogen interactions and pathogen transmissibility by ticks. Infection of hard ticks by artificial feeding was reported by other authors as well, emphasizing the efficiency of this method to successfully infect ticks with pathogens of viral, bacterial and parasitic origin [28,29,30,31]. This might also be a possible explanation for the different results between our study and that done by Lawrie et al. (2004). Ticks used in this study were exposed to higher viral titres (5.95 log_10_TCID50/mL in the artificial feeding system) than in the other study (viremic mice with 2–3 log_10_ PFU/mL).

When surveying for the tick-to-blood meal transmission by feeding of females, we did not detect WNV in the blood, leading to the assumption that *I. ricinus* ticks are not competent to transmit the virus while feeding on a host. In contrast to these results, one report showed that *Hyalomma marginatum* nymphs and females were able in fact to transmit the virus to uninfected hosts during feeding, concluding that this species could be involved in the natural transmission cycle of WNV [11]. Immature larval and nymphal stages of *H. marginatum* were infected by feeding on viremic rabbits. The infected ticks were allowed to molt into the next stages and, after feeding on naïve rabbits, WNV antibodies were detected by indirect immunofluorescence assay in the blood of the infested hosts. The putative vector competence of *H. marginatum* for WNV could be an outcome of the existent strong association between immature stages of this species and passerine birds, an association that is missing between birds and *I. ricinus* immature ticks. Larvae and nymphs of *H. marginatum* preferentially feed on birds (blackbird, thrush bird, great tit, common accentor, red tailed bird) rather than rodents [32,33], and the role that birds play as reservoir hosts for WNV could also involve *H. marginatum* ticks in the transmission cycle. These results place *H. marginatum* next to soft tick *Ornithodorus moubata,* for which the potential viral transmission to uninfected mice was also indicated in brain samples from one mouse infested with 20 infected soft ticks, with the brain samples testing positive for WNV RNA by RT-PCR but negative by immunofluorescence assay [12].

In order to be further transmitted by ticks during feeding, a viral agent must reach the salivary glands (SG) by overcoming, in addition to the innate immune system, several barriers (midgut infection barrier, midgut release barrier, midgut escape barrier, SG infection barrier, and SG release barrier) [34]. When it comes to the antiviral response of ticks, several pathways have been described as involved in the viral defense such as immune deficiency (IMD), RNA interference (RNAi), Toll and Janus kinase-signal transducers and activation of transcription (JAK/STAT) [35]. Of all the compartments and pathways involved in the antiviral response in ticks, the midgut seems to have the most important role in the survival and replication of the viral agents [36]. The digestion of blood by ticks is a specialized intracellular process that takes place in the cells of the midgut [7]. According to the results of this study, WNV was able to withstand the blood digestion from the midgut, persisting at low titres through the molting process of ticks. The incubation period of *I. ricinus* engorged nymphs that molted into adult males, which subsequently tested positive for WNV, totalized 53 days. This long period during which WNV can persist in ticks corroborated with the life cycle characteristics and longevity of these arthropods could indicate their potential role as reservoir hosts for the virus. Immature stages of *I. ricinus* (larvae and nymphs) frequently feed on resident and migratory birds, especially on ground-feeding birds [14], and can acquire WNV during feeding with blood on the natural hosts of this viral agent. In addition, this tick species is capable to feed on a broad range of hosts (more than 300 different vertebrates) [16], representing, as such, a connector between different eco-systems from which WNV could benefit. Infected immature tick stages could then maintain the virus during unfavorable climatic conditions for mosquito vectors and, as suggested by Anderson et al. (2004), could be ingested by birds, leading to a new infection with WNV as this route of viral acquisition was previously mentioned [37,38,39].

After carrying out artificial feeding to study transmission of WNV lineage 1 by ticks, we tested partially engorged *I. ricinus* females for WNV and, apart from two ticks, collected at days 3 and 4 of feeding, all samples tested negative for the virus. This low rate of transstadial persistence of WNV in adult females compared to males might be a result of the tick immune system activation during blood feeding. It could also indicate the inability of WNV to reach and infect the salivary glands prior the feeding of ticks. In contrast, it is known that tick-borne encephalitis virus and Powassan virus are able to reach salivary glands of ticks before the onset of feeding and, as a result, are successfully transmitted to the hosts at very early stages of feeding [40,41].

This study demonstrated that laboratory reared *Ixodes ricinus* nymphs can successfully be infected with West Nile virus lineage 1 by in vitro feeding and the virus persists transstadially in this species. Even with this persistence, ticks did not transmit WNV during feeding and it is unlikely that *I. ricinus* represents a competent vector for the virus. However, there is the potential reservoir role that this tick species can have, as it is able to maintain the viral agent for long periods of time compared to the natural vectors represented by mosquitoes.

## 4. Materials and Methods

### 4.1. Ethics Statement

The procedures for the feeding of laboratory tick colonies on rabbits and gerbils were approved by the State Office for Agriculture, Food Safety and Fishing of Mecklenburg-Western Pomerania (LALLF) (reference number: 7221.3-2-008/16). The experimental infection of ticks was done in accordance with the 3R principles of animal experimentation: replace, reduce and refine for scientific purposes.

### 4.2. Tick Colony Rearing

Adult laboratory reared *Ixodes ricinus* ticks (males and females) were obtained from Institute of Parasitology and Tropical Veterinary Medicine, Freie Universität, Berlin, Germany. Ticks were further reared in special designated tick-rearing facilities within the Institute of Infectology, Friedrich Loeffler Institut, in order to obtain the first new generation of *I. ricinus* nymphs that were used in all experiments. Briefly, adult stages of *I. ricinus* were fed on New Zealand white rabbits (obtained from the animal breeding facilities at Friedrich Loeffler Institut), by ear bag infestation, while larval and nymphal stages were fed on gerbils (Charles River Laboratories, Sulzfeld, Germany). During tick infestation, gerbils were held in cages with steel grid at the bottom and the cages placed inside a water pool. Between feeding and infection experiments, ticks were kept in an incubator at 22 °C with an atmosphere of 85–90% relative humidity (rH) and 16 h light/8 h dark cycle.

### 4.3. Virus

The WNV lineage 1 (Magpie/Italy/2008/203204, GenBank No. JF719066) was used for the experimental infection of *I. ricinus* ticks. In order to obtain the viral stocks, the virus was passaged on Vero cells (Collection of Cell Lines in Veterinary Medicine (CCLV), Friedrich-Loeffler-Institut, Insel Riems-Greifswald, Germany) in modified minimum essential medium (MEM) with 2% fetal calf serum (FCS). The virus was harvested at day 7 post infection (7 dpi) and the titre was determined on Vero cells by using the endpoint dilution assay and the Spearman–Kärber algorithm [42]. The titre of the virus stock used for injection of ticks was 7.75 log_10_ 50% tissue culture infective dose (TCID_50_) per mL while a viral stock with a titre of 6.25 log_10_ TCID50/mL was used for the infection of ticks by in vitro feeding. The virus was stored at −70 °C in aliquots of 1 mL.

### 4.4. Infection of Ticks by Percoxal Injection

In order to study the viral replication kinetics of WNV, unfed *I. ricinus* nymphs were infected by percoxal route with 69 nL of approximately 3.59 log_10_TCID50. In total, 98 *Ixodes ricinus* nymphs were injected into the haemocel through the coxal plate of the fourth pair of legs (Video S1). The injection of ticks was performed using a digital microinjector system (Nanoject II, Drummond Scientific, Broomall, PA, USA) and 5 µL fine-pulled capillaries in a glovebox (Terra Universal, Fullerton, CA, USA) by placing ticks on a double sided sticky tape with the ventral side up and exposing the coxal plate with fine forceps (Video S1). In addition, 12 nymphs were injected with the identical quantity of clear MEM as controls. After injection, ticks were kept in an incubator at 22 °C, 90% rH and 16 h light/8 h dark cycle for 28 days. During the incubation period, injected ticks were sampled at different timepoints (days 0, 7, 14, 21, 28), with four ticks per timepoint being analyzed (except for day 28, with three ticks available). After sampling, ticks were stored at −70 °C until further processing.

### 4.5. In Vitro Feeding System

The artificial feeding system used in the current work was built based on the protocols published by Kröber and Guerin [43] and Krull et al. [44]. In short, lens cleaning papers covered with a mixture of silicone, white colour paste (Wacker Chemie, Munich, Germany), silicone oil and hexane (Carl Roth GmbH, Karlsruhe, Germany), were used as the feeding membranes. Membranes thickness was measured with a micro calliper; those with a thickness of 50–60 µm were used for feeding *I. ricinus* nymphs while membranes with a thickness of 80–100 µm were used to feed adult females. The membranes were glued, using Elastosil E41 (Wacker Chemie, Munich, Germany) to acrylic glass tubes (Hbholzmaus, Uedem, Germany) of 5 cm length, 2.6 cm inner diameter and 3 cm outer diameter. The membranes were then checked for leakage and surface sterilised by placing them in a 6-well plate (Sarstedt, Nuembrecht, Germany) with 70% ethanol for 30 min. On the membranes used for feeding *I. ricinus* females, a square piece of mosquito netting material was glued for adding tactile stimuli. Bovine hair extract was obtained by soaking chopped hair of approximately 1 cm length in a beaker with 10 mL of methanol (Carl Roth GmbH, Karlsruhe, Germany) and left at room temperature for at least 4 days to allow alcohol evaporation [30]. One hour prior the beginning of tick feeding, 100 µL of hair perfume was added to each membrane and left to dry at room temperature, followed by the addition of conspecific tick faeces and bovine hair as extra attachment stimuli.

### 4.6. Artificial Tick Feeding

Artificial feeding of *I. ricinus* nymphs was done to evaluate the transstadial transmission of WNV while in vitro feeding of molted female ticks was performed to study the potential virus transmission from ticks to the blood during feeding. For the infection of nymphs with WNV, four feeding units (hereafter FUs) each containing 30 nymphs and one *I. ricinus* female were used, while one FU with 30 nymphs and one female served as negative control. Adult female ticks were added to each FU as stimuli for nymphs to attach to the membrane (Figure S1).

The FUs containing the ticks were placed on 6-well plates, with each well containing pre-warmed blood at 37 °C by placing the 6-well plates on a heating plate (W10 heating plate, VWR, Hannover, Germany). The artificial feeding system was kept in an incubator (MLR-352H-PE; Panasonic Corporation, Osaka, Japan) under controlled conditions (22 °C, rH of 90%, 16 h light: 8 h dark photo cycle). Both nymphs and females were fed with sterile heparinized bovine blood (Fiebig-Nährstofftechnik GbR, Idstein, Germany) supplemented with 4 g/L glucose (Carl Roth GmbH, Karlsruhe, Germany) by adding 3.03 mL blood meal for each FU.

For infection of nymphs with WNV, each FU belonging to the experimental group was placed on blood meal containing 2.5 mL bovine heparinized blood, 500 µL of the virus stock with a titre of 6.25 log_10_TCID50/mL and 30 µL of ATP 0.1 M (Merck, St. Louis, MO, USA) as a phagostimulant, the viral titre of the blood meal being approx. 5.95 log_10_TCID50/mL. The spike of the blood meal with WNV was done after 24 h from the debut of tick feeding, to allow nymphs to attach and have an active feeding process. The infectious blood meal was maintained during four consecutive days, being changed twice a day at 8 and 16 h intervals. The infectious blood meal was titrated before and after feeding on Vero cells for confirmation of the viral titre.

The composition of uninfected blood meal consisted of 3 mL bovine heparinized blood and 30 µL ATP 0.1 M. During artificial feeding with non-infectious blood meal, changing of blood was done once every 24 h. Feeding of female ticks that molted from the nymphs of the experimental group was done by placing one individual tick per FU together with a laboratory-reared uninfected male. During the entire feeding process of *I. ricinus* females, blood meal samples were collected at 24 h intervals (100 µL for inoculation of Vero cells and 100 µL for detection of WNV RNA).

### 4.7. Ticks and Blood Samples Processing

In order to analyse tick samples for the presence of WNV, each tick was homogenized in a 2 mL tube containing 200 µL of MEM and one 5 mm sterile steel bead. Homogenization of ticks was done using Tissue Lyser II (Qiagen, Hilden, Germany) twice for 1 min, at 30 Hz. A volume of 100 µL of the tick homogenates was transferred to a 1.5 mL tube containing TRIZOL (Invitrogen, Karlsruhe, Germany) reagent for total RNA extraction and stored at −70 °C until further processing. The remaining 100 µL of the homogenate were added on Vero cells layer seeded in a 96-well-plate for the isolation of infectious virus. The cells were kept in MEM with 2% FCS and antimicrobials (penicillin 100 U/mL, streptomycin 100 µg/mL, gentamycin 50 mg/mL, amphotericin B 1 mg/L). Vero cells were incubated at 37 °C and 5% CO_2_ atmosphere for seven days and prior formalin inactivation of the 96-well-plate, 100 µL of the cell supernatants were collected from all samples, regardless of the cytopathic effect (CPE) and placed in TRIZOL reagent for RNA extraction.

To study the vector competence of *I. ricinus* females of the experimental group, 200 µL of blood meal was collected daily during in vitro feeding, following a similar protocol as for the tick homogenates with the exception that only 50 µL of blood meal were added on Vero cells monolayer and incubated until day 7. For the isolation of total RNA, 100 µL of blood meal and Vero cell supernatants were placed in TRIZOL LS (Invitrogen, Karlsruhe, Germany) and TRIZOL reagent, respectively.

Female ticks were allowed to feed until repletion and to lay eggs for the evaluation of the transovarial persistence of WNV. Egg batches and the females that laid eggs were processed similarly as tick samples but underwent only RT-qPCR analysis for the identification of WNV RNA.

Total RNA extraction of all samples was done by initial phase separation using TRIZOL reagent, then the obtained aqueous phase was further processed for an automatic RNA extraction by using NucleoMag^®^ VET kit (Macherey-Nagel, Düren, Germany) and the King Fisher^®^ Flex Purification system (ThermoFisher, Darmstedt, Germany), following the manufacturer’s instructions. Total RNA was eluted in 100 µL of elution buffer then stored at −70 °C until further use.

### 4.8. Molecular Detection of WNV RNA

Amplification of RNA extracted from tick samples and blood meal was done by RT-qPCR targeting the non-structural NS2A region of WNV genome, using iTaq^TM^ Universal Probes One Step Kit (BioRad Laboratory Inc., Munich, Germany) and CFX-96 Real-Time system (BioRad Laboratory Inc., Munich, Germany) as previously described [45]. The samples were tested in duplicates and, for the quantification of relative viral titres, tenfold-dilution series of WNV RNA were run in parallel as standards, each dilution point having three replicates (Table S3, Figures S2 and S3). Standards used in the RT-qPCR reaction were obtained from WNV viral stocks with a defined titre (TCID50/mL), by extracting 100 µL of viral aliquots with TRIZOL reagent, according to the manufacturers’ instructions.

### 4.9. Statistical Analysis

Graphical presentations were built using R version 3.6.2 in R Studio [46,47]. One-tailed *t*-test was done using GraphPad Prism version 8.1.0 for Windows (GraphPad Software, La Jolla, CA, USA, www.graphpad.com) for the comparison of viral titres between WNV injected nymph groups collected at different timepoints post-infection, with differences being considered significant at *p*-values lower than 0.05.

## Figures and Tables

**Table 1:** In vitro feeding for the virus transmission study of *Ixodes ricinus* females infected with WNV lineage 1 during nymphal stage.

FUs	Feeding Status															Engorged Ticks
	D1	D2	D3	D4	D5	D6	D7	D8	D9	D10	D11	D12	D13	D14	D15	
FU1-1	N	N	N	N	F	F	F	F	F	N	X	X	X	X	X	0
FU1-2	N	N	N	N	N	N	N	N	N	X	X	X	X	X	X	0
FU1-3	N	N	N	F	F	F	F	F	F	F	F	F	H			1 ^a^
FU1-4	F	F	F	F	F	F	F	F	N	N	N	N	H			1 ^a^
FU2-1	F	F	F	F	F	F	F	F	N	N	N	N	H			1 ^b^
FU2-2	N	X	X	X	X	X	X	X	X	X	X	X	X	X	X	0
FU2-3	N	N	F	F	F	F	N	F	F	F	F	F	F	N	H	1 ^a^
FU2-4	N	N	N	F	F	F	F	F	F	F	X	X	X	X	X	0
FU2-5	N	F	F	F	F	F	F	F	F	H						1 ^b^
FU2-6	F	N	X	X	X	X	X	X	X	X	X	X	X	X	X	0
FU3-1	F	F	F	F	F	F	F	F	F	F	H					1 ^a^
FU3-2	N	N	N	N	N	F	F	F	F	F	N	N	H			1 ^b^
FU3-3	N	N	N	N	N	N	F	N	N	X	X	X	X	X	X	0
FU3-4	F	F	F	F	X	X	X	X	X	X	X	X	X	X	X	0
FU3-5	N	N	F	F	F	X	X	X	X	X	X	X	X	X	X	0
FU3-6	F	F	F	X	X	X	X	X	X	X	X	X	X	X	X	0
FU3-7	N	N	N	F	F	F	F	F	F	X	X	X	X	X	X	0
FU4-1	F	F	F	X	X	X	X	X	X	X	X	X	X	X	X	0
FU4-2	F	F	F	F	F	F	F	N	N	N	H					1 ^a^
FU4-3	N	N	N	N	N	N	N	N	N	X	X	X	X	X	X	0
FU4-4	N	N	N	F	F	N	X	X	X	X	X	X	X	X	X	0
FU4-5	N	N	N	N	X	X	X	X	X	X	X	X	X	X	X	0
FU4-6	N	N	F	F	F	F	F	F	F	F	H					1
Total	8	8	11	13	13	12	12	10	9	6	2	2	1	0	0	9
Neg. control *	3	3	3	4	4	5	4	5	5	5	3	3	1	1	H	3 ^c^

FUs: feeding units containing one *Ixodes ricinus* female from the experimental group; D1–D15: days of in vitro feeding; N: not feeding; F: time point when female ticks were observed to be attached to the membrane; X: feeding cancelled; H: day when engorged ticks were harvested; * Negative control FU with 6 females; ^a^ Engorged ticks that died during incubation; ^b^ Females that laid eggs; ^c^ Two out of three females from the control group laid eggs.

## Supplementary Materials

The following are available online at https://www.mdpi.com/2076-0817/9/10/780/s1, Table S1: WNV RNA detection in tick homogenates and Vero cell supernatants of Ixodes ricinus nymphs infected by injection and collected at different time points post infection, Table S2: WNV RNA detection in tick homogenates and Vero cell supernatants of Ixodes ricinus males fed on infectious blood meal during the nymphal stage, Table S3: WNV RNA standards obtained from WNV viral stocks with a defined titre (TCID_50_/mL) used for relative quantification of WNV Lin. 1 in ticks, Figure S1: Artificial feeding of nymphs, Figure S2: Amplification cycles of tenfold-dilution series of WNV RNA standards, Figure S3: Standard curve of tenfold-dilution series of WNV RNA standards, Video S1: Percoxal injection of a nymph.

## References

[1] Chancey C., Grinev A., Volkova E., Rios M. (2015). The global ecology and epidemiology of West Nile virus. Biomed. Res. Int..

[2] Campbell G.L., Marfin A.A., Lanciotti R.S., Gubler D.J. (2002). West Nile virus. Lancet Infect. Dis..

[3] Kolodziejek J., Marinov M., Kiss B.J., Alexe V., Nowotny N. (2014). The complete sequence of a West Nile virus lineage 2 strain detected in a *Hyalomma marginatum marginatum* tick collected from a song thrush (*Turdus philomelos*) in eastern Romania in 2013 revealed closest genetic relationship to strain Volgograd 2007. PLoS ONE.

[4] Hoogstraal H., Clifford C.M., Keirans J.E., Kaiser M.N., Evans D.E. (1976). The *Ornithodoros* (*Alectorobius*) *capensis* group (Acarina: Ixodoidea: Argasidae) of the palearctic and oriental regions. *O*. (*A*.) *maritimus*: Identity, marine bird hosts, virus infections, and distribution in western Europe and northwestern Africa. J. Parasitol..

[5] Lvov D.K., Timopheeva A.A., Smirnov V.A., Gromashevsky V.L., Sidorova G.A., Nikiforov L.P., Sazonov A.A., Andreev A.P., Skvortzova T.M., Beresina L.K. (1975). Ecology of tick-borne viruses in colonies of birds in the USSR. Med.Biol..

[6] Lwande O.W., Lutomiah J., Obanda V., Gakuya F., Mutisya J., Mulwa F., Michuki G., Chepkorir E., Fischer A., Venter M. (2013). Isolation of tick and mosquito-borne arboviruses from ticks sampled from livestock and wild animal hosts in Ijara District, Kenya. Vector Borne Zoonotic Dis..

[7] Platonov A.E. (2001). West Nile encephalitis in Russia 1999-2001: Were we ready? Are we ready?. Ann. N. Y. Acad. Sci..

[8] Flores F.S., Zanluca C., Guglielmone A.A., Duarte Dos Santos C.N., Labruna M.B., Diaz A. (2019). Vector competence for West Nile virus and St. Louis Encephalitis virus (Flavivirus) of three tick species of the genus *Amblyomma* (Acari: Ixodidae). Am. J. Trop. Med. Hyg..

[9] Anderson J.F., Main A.J., Andreadis T.G., Wikel S.K., Vossbrinck C.R. (2003). Transstadial transfer of West Nile virus by three species of ixodid ticks (Acari: Ixodidae). J. Med. Entomol..

[10] Reisen W.K., Brault A.C., Martinez V.M., Fang Y., Simmons K., Garcia S., Omi-Olsen E., Lane R.S. (2007). Ability of transstadially infected *Ixodes pacificus* (Acari: Ixodidae) to transmit West Nile virus to song sparrows or western fence lizards. J. Med. Entomol..

[11] Formosinho P., Santos-Silva M.M. (2006). Experimental infection of *Hyalomma marginatum* ticks with West Nile virus. Acta Virol..

[12] Lawrie C.H., Uzcátegui N.Y., Gould E.A., Nuttall P.A. (2004). Ixodid and argasid tick species and West Nile virus. Emerg. Infect. Dis..

[13] Whitman L., Aitken T.H. (1960). Potentiality of *Ornithodoros moubata* Murray (Acarina, Argasidae) as a reservoir vector of West Nile virus. Ann. Trop. Med. Parasitol..

[14] Rizzoli A., Silaghi C., Obiegala A., Rudolf I., Hubálek Z., Földvári G., Plantard O., Vayssier-Taussat M., Bonnet S., Špitalská E. (2014). *Ixodes ricinus* and its transmitted pathogens in urban and peri-urban areas in Europe: New hazards and relevance for public health. Front. Public Health.

[15] Randolph S.E. (2009). Tick-borne disease systems emerge from the shadows: The beauty lies in molecular detail, the message in epidemiology. Parasitology.

[16] Medlock J.M., Hansford K.M., Bormane A., Derdakova M., Estrada- Peña A., George J.C., Golovljova I., Jaenson T.G., Jensen J.K., Jensen P.M. (2013). Driving forces for changes in geographical distribution of *Ixodes ricinus* ticks in Europe. Parasites Vectors.

[17] Hai V.V., Almeras L., Socolovschi C., Raoult D., Parola P., Pagès F. (2014). Monitoring human tick-borne disease risk and tick bite exposure in Europe: Available tools and promising future methods. Ticks Tick Borne Dis..

[18] Gritsun T.S., Lashkevich V.A., Gould E.A. (2003). Tick-borne encephalitis. Antivir. Res..

[19] Rizzoli A., Hauffe H., Carpi G., Vourc’h G.I., Neteler M., Rosà R. (2011). Lyme borreliosis in Europe. EuroSurveillance.

[20] Rockstroh A., Moges B., Berneck B.S., Sattler T., Revilla- Fernández S., Schmoll F., Pacenti M., Sinigaglia A., Barzon L., Schmidt-Chanasit J. (2019). Specific detection and differentiation of tick-borne encephalitis and West Nile virus induced IgG antibodies in humans and horses. Transbound. Emerg. Dis..

[21] Norte A.C., de Carvalho I.L., Ramos J.A., Gonçalves M., Gern L., Núncio M.S. (2012). Diversity and seasonal patterns of ticks parasitizing wild birds in western Portugal. Exp. Appl. Acarol..

[22] Camp J.V., Nowotny N. (2020). The knowns and unknowns of West Nile virus in Europe: What did we learn from the 2018 outbreak?. Expert Rev. Anti Infect. Ther..

[23] European Centre for Disease Prevention and Control (ECDC) (2019). Epidemiological Update: West Nile Virus Transmission Season in Europe. https://www.ecdc.europa.eu/en/news-events/epidemiological-update-west-nile-virus-transmission-season-europe-2019.

[24] Ziegler U., Lühken R., Keller M., Cadar D., van der Grinten E., Michel F., Albrecht K., Eiden M., Rinder M., Lachmann L. (2019). West Nile virus epizootic in Germany, 2018. Antiviral. Res..

[25] European Centre for Disease Prevention and Control (ECDC) Tick Maps [Internet]. https://ecdc.europa.eu/en/disease-vectors/surveillance-and-disease-data/tick-maps.

[26] Rechav Y., Zyzak M., Fielden L.J., Childs J.E. (1999). Comparison of methods for introducing and producing artificial infection of ixodid ticks (Acari: Ixodidae) with *Ehrlichia chaffeensis*. J. Med. Entomol..

[27] Ličková M., Fumacova Havlíková S., Sláviková M., Slovák M., Drexler J.F., Klempa B. (2020). *Dermacentor reticulatus* is a vector of tick-borne encephalitis virus. Ticks Tick Borne Dis..

[28] Vimonish R., Johnson W.C., Mousel M.R., Brayton K.A., Scoles G.A., Noh S.M., Ueti M.W. (2020). Quantitative analysis of *Anaplasma marginale* acquisition and transmission by *Dermacentor andersoni* fed in vitro. Sci. Rep..

[29] Körner S., Makert G.R., Mertens-Scholz K., Henning K., Pfeffer M., Starke A., Nijhof A.M., Ulbert S. (2020). Uptake and fecal excretion of *Coxiella burnetii* by *Ixodes ricinus* and *Dermacentor marginatus* ticks. Parasites Vectors.

[30] Tajeri S., Razmi G., Haghparast A. (2016). Establishment of an artificial tick feeding system to study *Theileria lestoquardi* infection. PLoS ONE.

[31] Bouwknegt C., van Rijn P.A., Schipper J.J., Hölzel D., Boonstra J., Nijhof A.M., van Rooij E.M., Jongejan F. (2010). Potential role of ticks as vectors of bluetongue virus. Exp. Appl. Acarol..

[32] Valcárcel F., González J., González M.G., Sánchez M., Tercero J.M., Elhachimi L., Carbonell J.D., Olmeda A.S. (2020). Comparative Ecology of *Hyalomma lusitanicum* and *Hyalomma marginatum* Koch, 1844 (Acarina: Ixodidae). Insects.

[33] Kotti B.K., Shaposhnikova L.I., Evchenko Iu M., Levchenko B.I., Surkhaev D.B., Korzhov P.N., Tokhov Iu M. (2001). [*Hyalomma marginatum* Koch in Stavropol’ region]. Zhurnal Mikrobiol. Epidemiol. Immunobiol..

[34] Nuttall P.A. (2014). Tick-Borne Viruses.

[35] Rückert C., Bell-Sakyi L., Fazakerley J.K., Fragkoudis R. (2014). Antiviral responses of arthropod vectors: An update on recent advances. Virus Dis..

[36] Hajdušek O., Šíma R., Ayllón N., Jalovecká M., Perner J., de la Fuente J., Kopáček P. (2013). Interaction of the tick immune system with transmitted pathogens. Front. Cell. Infect. Microbiol..

[37] Garmendia A.E., Van Kruiningen H.J., French R.A., Anderson J.F., Andreadis T.G., Kumar A., West A.B. (2000). Recovery and identification of West Nile virus from a hawk in winter. J. Clin. Microbiol..

[38] Odelola H.A., Oduye O.O. (1977). West Nile virus infection of adult mice by oral route. Arch. Virol..

[39] Komar N., Langevin S., Hinten S., Nemeth N., Edwards E., Hettler D., Davis B., Bowen R., Bunning M. (2003). Experimental infection of North American birds with the New York 1999 strain of West Nile virus. Emerg. Infect. Dis..

[40] Chernesky M.A., McLean D.M. (1969). Localization of Powassan virus in *Dermacentor andersoni* ticks by immunofluorescence. Can. J. Microbiol..

[41] Řeháček J. (1965). Development of animal viruses and rickettsiae in ticks and mites. Annu. Rev. Entomol..

[42] Ramakrishnan M.A. (2016). Determination of 50% endpoint titer using a simple formula. World J. Virol..

[43] Kröber T., Guerin P.M. (2007). In vitro feeding assays for hard ticks. Trends Parasitol..

[44] Krull C., Böhme B., Clausen P.H., Nijhof A.M. (2017). Optimization of an artificial tick feeding assay for *Dermacentor reticulatus*. Parasites Vectors.

[45] Eiden M., Vina-Rodriguez A., Hoffmann B., Ziegler U., Groschup M.H. (2010). Two new real-time quantitative reverse transcription polymerase chain reaction assays with unique target sites for the specific and sensitive detection of lineages 1 and 2 West Nile virus strains. J. Vet. Diagn. Investig..

[46] R Core Team (2019). R: A Language and Environment for Statistical Computing.

[47] RStudio Team (2019). RStudio: Integrated Development for R.

