# Supplementary material for: Transstadial Transmission and Replication Kinetics of West Nile Virus Lineage 1 in Laboratory Reared Ixodes ricinus Ticks
Source: Pathogens. 2020 Sep 24;9(10):780. doi: 10.3390/pathogens9100780 (PMC7650586; doi:10.3390/pathogens9100780)
Supplement: Supplementary file 1 [file pathogens-09-00780-s001.zip › pathogens-935443-Supplementary - Final Version/pathogens-935443-Supplmentary File-Final Version.docx]

**Table S1.** WNV RNA detection in tick homogenates and Vero cell supernatants of *Ixodes ricinus* nymphs infected by injection and collected at different time points post infection.

| **Sample ID** | **Collection Point** | **Ct Value in Nymphs** | **Estimated log_10_TCID_50_ in Nymphs** | **Ct Value in Vero Supernatants** | **Estimated log_10_TCID_50_ in Vero Supernatants** |
| --- | --- | --- | --- | --- | --- |
| AD0-1 | 0 dpi | 27.82 | 3.30 | 35.35 | 1.14 |
| AD0-2 | 0 dpi | 26.13 | 3.78 | 32.15 | 2.05 |
| AD0-3 | 0 dpi | 27.79 | 3.31 | 16.62 | 6.52 |
| AD0-4 | 0 dpi | 27.82 | 3.30 | 33.70 | 1.61 |
| AD7-1 | 7 dpi | 29.26 | 2.88 | 17.93 | 6.14 |
| AD7-2 | 7 dpi | 30.57 | 2.51 | 34.74 | 1.32 |
| AD7-3 | 7 dpi | 30.61 | 2.50 | 34.98 | 1.28 |
| AD7-4 | 7 dpi | 27.99 | 3.25 | 14.39 | 7.16 |
| AD14-1 | 14 dpi | 27.68 | 3.34 | 14.95 | 7.00 |
| AD14-2 | 14 dpi | 24.25 | 4.32 | 15.49 | 7.14 |
| AD14-3 | 14 dpi | 29.34 | 2.86 | 16.78 | 6.78 |
| AD14-4 | 14 dpi | 30.62 | 2.49 | 39.23 | 0.60 |
| AD21-1 | 21 dpi | 27.22 | 3.47 | 37.89 | 0.93 |
| AD21-2 | 21 dpi | 29.85 | 2.71 | 33.92 | 2.02 |
| AD21-3 | 21 dpi | 22.02 | 4.96 | 33.16 | 2.23 |
| AD21-4 | 21 dpi | 25.74 | 3.91 | 16.07 | 6.98 |
| AD28-1 | 28 dpi | 22.24 | 4.90 | 16.06 | 6.98 |
| AD28-2 | 28 dpi | 23.80 | 4.46 | 15.20 | 7.22 |
| AD28-3 | 28 dpi | 31.22 | 2.32 | 32.21 | 2.49 |

dpi: days post infection; Ct: cycle threshold; log_10_TCID_50_: log_10_ 50% tissue culture infective dose per mL

**Table S2.** WNV RNA detection in tick homogenates and Vero cell supernatants of *Ixodes ricinus* males fed on infectious blood meal during the nymphal stage.

| **Sample ID** | **Ct Value in Male Ticks** | **Estimated**  **log_10_ TCID_50_**  **in Male Ticks** | **Ct Value in Vero Supernatants** | **Estimated**  **log_10_ TCID_50_ in**  **Vero Supernatants** |
| --- | --- | --- | --- | --- |
| FU1-1 | 36.20 | 0.71 | 36.29 | 0.68 |
| FU1-2 | 33.08 | 1.58 | 33.61 | 1.42 |
| FU1-3 | - | - | 36.11 | 0.73 |
| FU1-4 | 38.69 | 0.01 | 35.05 | 1.02 |
| FU1-5 | 38.63 | 0.02 | 35.59 | 0.87 |
| FU1-6 | 31.12 | 2.12 | 36.40 | 0.65 |
| FU1-7 | 33.51 | 1.45 | 36.01 | 0.76 |
| FU1-8 | 32.65 | 1.69 | - | - |
| FU1-9 | 34.28 | 1.24 | - | - |
| FU1-10 | 33.15 | 1.56 | 37.70 | 0.29 |
| FU1-11 | 31.57 | 1.99 | - | - |
| FU1-13 | 32.56 | 1.72 | 38.44 | 0.08 |
| FU2-1 | 33.34 | 1.50 | - | - |
| FU2-3 | - | - | 36.59 | 0.60 |
| FU2-4 | 34 | 1.32 | 35.19 | 0.99 |
| FU2-5 | 32.06 | 1.86 | 34.65 | 1.14 |
| FU2-6 | 34.40 | 1.21 | 37.34 | 0.38 |
| FU2-7 | - | - | 34.80 | 1.09 |
| FU2-8 | - | - | 36.60 | 0.59 |
| FU3-1 | 34.53 | 1.17 | 35.57 | 0.88 |
| FU3-2 | 32.48 | 1.74 | 35.01 | 1.04 |
| FU4-1 | 35.69 | 0.85 | - | - |
| FU4-2 | 34.08 | 1.30 | - | - |
| FU4-4 | 36.89 | 0.51 | - | - |
| FU4-6 | - | - | 36.25 | 0.69 |
| FU4-7 | 37.46 | 0.35 | 36.41 | 0.65 |

Ct: cycle threshold; log_10_TCID_50_: log_10_ 50% tissue culture infective dose per mL.

**Table S3.** WNV RNA standards obtained from WNV viral stocks with a defined titre (TCID_50_/mL) used for relative quantification of WNV Lin. 1 in ticks.

| **Standard ID** | **Fluor** | **Target** | **Content** | **Ct value** | **Estimated log_10_TCID_50_** |
| --- | --- | --- | --- | --- | --- |
| Std-1 | FAM | NS2A | Std | 19.46 | 5.50 |
| Std-1 | FAM | NS2A | Std | 19.39 | 5.50 |
| Std-1 | FAM | NS2A | Std | 19.84 | 5.50 |
| Std-2 | FAM | NS2A | Std | 23.59 | 4.50 |
| Std-2 | FAM | NS2A | Std | 23.51 | 4.50 |
| Std-2 | FAM | NS2A | Std | 23.41 | 4.50 |
| Std-3 | FAM | NS2A | Std | 27.06 | 3.50 |
| Std-3 | FAM | NS2A | Std | 26.67 | 3.50 |
| Std-3 | FAM | NS2A | Std | 27.03 | 3.50 |
| Std-4 | FAM | NS2A | Std | 31.01 | 2.50 |
| Std-4 | FAM | NS2A | Std | 30.99 | 2.50 |
| Std-4 | FAM | NS2A | Std | 30.66 | 2.50 |
| Std-5 | FAM | NS2A | Std | 34.26 | 1.50 |
| Std-5 | FAM | NS2A | Std | 34.25 | 1.50 |
| Std-5 | FAM | NS2A | Std | 34.04 | 1.50 |
| Std-6 | FAM | NS2A | Std | 37.35 | 0.50 |
| Std-6 | FAM | NS2A | Std | 37.55 | 0.50 |
| Std-6 | FAM | NS2A | Std | 37.16 | 0.50 |

Std: standard; Ct: cycle threshold; log_10_TCID_50_: log_10_ 50% tissue culture infective dose per Ml


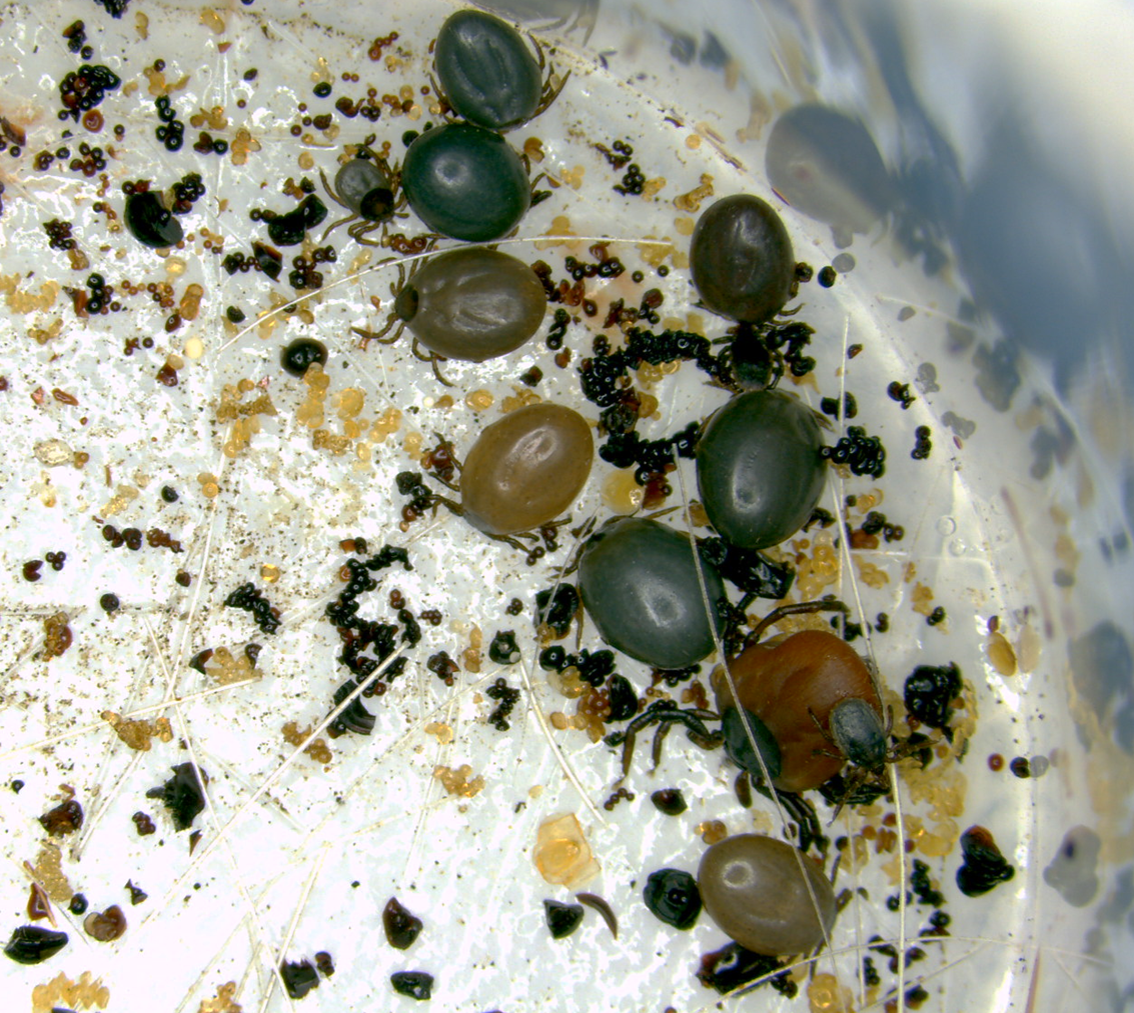


**Figure S1.** Artificial feeding of nymphs.


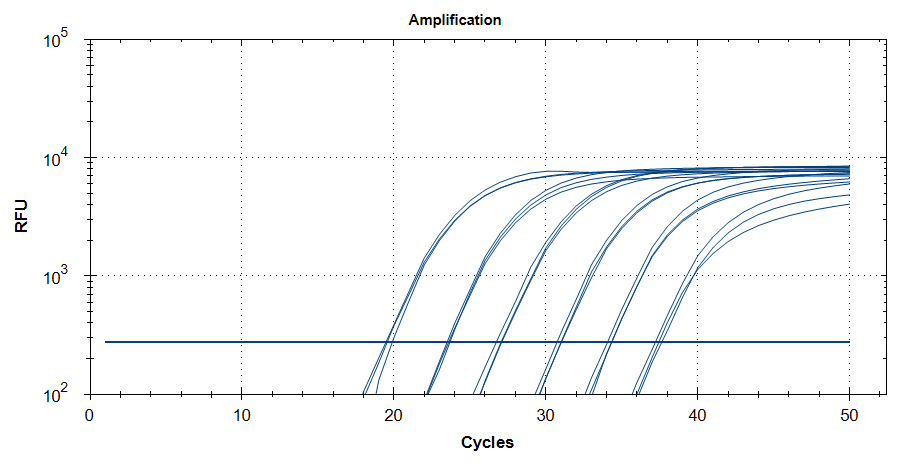


**Figure S2.** Amplification cycles of tenfold-dilution series of WNV RNA standards.

For the quantification of relative viral titres of samples, tenfold-dilution series of WNV RNA were run in parallel as standards (10^-1^ to 10^-6^ dilution series), each dilution point having three replicates. Standards of the RT-qPCR reaction were obtained from WNV viral stocks with a defined titre (TCID50/mL), by extracting RNA from 100 µL of viral aliquots with TRIZOL reagent, according to the manufacturers’ instructions.

**
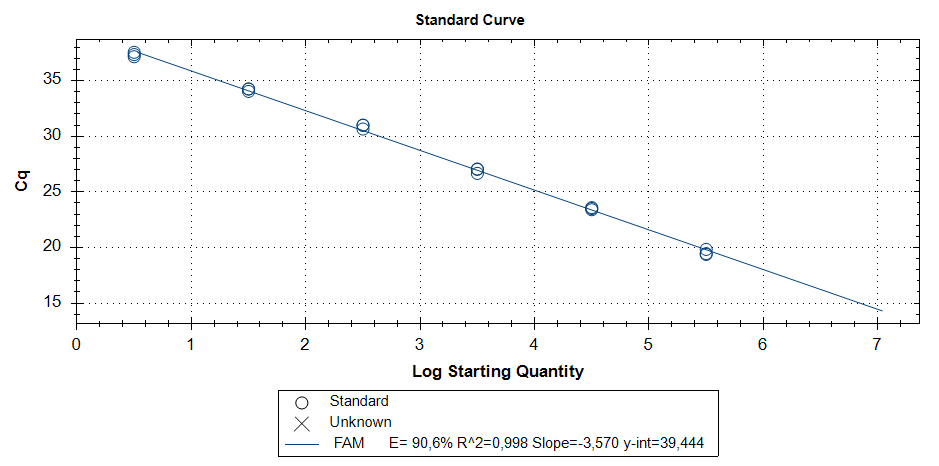
**

**Figure S3.** Standard curve of tenfold-dilution series of WNV RNA standards.

Standard curve of the tenfold-dilution series of WNV RNA standards that were run in parallel with the samples for relative quantification of WNV.
